# Extraction of Moso Bamboo Parameters Based on the Combination of ALS and TLS Point Cloud Data

**DOI:** 10.3390/s24134036

**Published:** 2024-06-21

**Authors:** Suying Fan, Sishuo Jing, Wenbing Xu, Bin Wu, Mingzhe Li, Haochen Jing

**Affiliations:** 1 State Key Laboratory of Subtropical Silviculture, Zhejiang A & F University, Hangzhou 311300, China; fffansying@stu.zafu.edu.cn (S.F.); 2021103021023@zafu.edu.cn (S.J.); leemingzhe@yeah.net (M.L.); jinghaochen2007@163.com (H.J.); 2 Key Laboratory of Carbon Cycling in Forest Ecosystems and Carbon Sequestration of Zhejiang Province, Zhejiang A & F University, Hangzhou 311300, China; 3 School of Environmental and Resources Science, Zhejiang A & F University, Hangzhou 311300, China; 4 School of Mathematics and Computer Science, Zhejiang A & F University, Hangzhou 311300, China; wubin@zafu.edu.cn

**Keywords:** ALS, TLS, moso bamboo, parameter extraction, point cloud alignment

## Abstract

Extracting moso bamboo parameters from single-source point cloud data has limitations. In this article, a new approach for extracting moso bamboo parameters using airborne laser scanning (ALS) and terrestrial laser scanning (TLS) point cloud data is proposed. Using the field-surveyed coordinates of plot corner points and the Iterative Closest Point (ICP) algorithm, the ALS and TLS point clouds were aligned. Considering the difference in point distribution of ALS, TLS, and the merged point cloud, individual bamboo plants were segmented from the ALS point cloud using the point cloud segmentation (PCS) algorithm, and individual bamboo plants were segmented from the TLS and the merged point cloud using the comparative shortest-path (CSP) method. The cylinder fitting method was used to estimate the diameter at breast height (DBH) of the segmented bamboo plants. The accuracy was calculated by comparing the bamboo parameter values extracted by the above methods with reference data in three sample plots. The comparison results showed that by using the merged data, the detection rate of moso bamboo plants could reach up to 97.30%; the R^2^ of the estimated bamboo height was increased to above 0.96, and the root mean square error (RMSE) decreased from 1.14 m at most to a range of 0.35–0.48 m, while the R^2^ of the DBH fit was increased to a range of 0.97–0.99, and the RMSE decreased from 0.004 m at most to a range of 0.001–0.003 m. The accuracy of moso bamboo parameter extraction was significantly improved by using the merged point cloud data.

## 1. Introduction

Moso bamboo grows fast and has a strong carbon sequestration capacity. It is a special forest resource with high economic and ecological value [1,2,3,4]. The structural characteristics of individual moso bamboo plants, such as the number of plants, diameter at breast height (DBH), moso bamboo height, crown width, etc., can be derived to calculate the biomass of moso bamboo [5,6,7]. In precision forestry, accurate extraction of moso bamboo parameters, such as bamboo height and DBH, can help to better evaluate the productivity of moso bamboo forests and manage them more rationally [8].

The traditional method for obtaining forest parameters is in-field survey, but this method has many problems, such as a long operation cycle, low efficiency, and high labor intensity. Therefore, it is not suitable for large-area forest surveys [9]. In recent years, airborne laser scanning (ALS) and terrestrial laser scanning (TLS), which are rapidly developing active remote sensing techniques, have been widely used in the acquisition of forest information [10], e.g., individual tree height, crown width, and DBH [11,12,13]. Both ALS and TLS are integral components of Light Detection and Ranging (LiDAR) technology. LiDAR is an active remote sensing technology that acquires three-dimensional information about a target structure by emitting and detecting laser pulses. This technique offers exceptional canopy penetration, high range resolution, and outstanding anti-interference capabilities [14,15,16].

A key step in individual tree-level studies is to identify and segment individual trees from point clouds. The individual tree segmentation algorithms based on ALS data can be mainly categorized into two groups, i.e., tree segmentation based on the canopy height model (CHM) and tree segmentation based on the point cloud [17]. The point cloud-based methods either segment tree crowns using the gaps between crowns [18] or detect trunks first and then segment the tree crowns [19]. However, ALS acquires data using a top-down scanning mode; hence, the laser points concentrate on the canopy layer. Due to the lack of laser points on tree trunks, trunk-based methods are less commonly adopted. In contrast, TLS adopts a bottom-up scanning mode, and tree trunks have denser laser points. Therefore, individual tree segmentation methods based on TLS data commonly detect tree trunks first [20,21,22,23].

Numerous studies on tree-level parameter extraction have been conducted in temperate, subtropical, and tropical forests. Bamboo forests are usually characterized by sparse understory vegetation, an overlapping canopy, and group growth. Studies on single bamboo parameter extraction are few. Li et al. [1] investigated the structure and dynamics of moso bamboo forests after reforestation by using TLS. Jiang et al. [24] performed single bamboo segmentation of moso bamboo based on TLS using the hierarchical distance discrimination method and estimated the biomass of individual bamboo plants based on the DBH and bamboo culm length extracted from the point cloud. Cao et al. [7] used ALS data to estimate bamboo forest canopy structure and biomass based on moso bamboo DBH and culm height.

Most studies have used only ALS or TLS data for the extraction of individual tree parameters, such as tree height and DBH [25,26]. However, the disparate scanning modes of ALS and TLS lead to differences in point distribution characteristics between ALS and TLS data. Therefore, how to merge ALS and TLS data to employ the complementary characteristics of these two data sets has become one of the key issues in current forest research [9].

Since ALS and TLS data are usually in different coordinate systems, point cloud alignment needs to be performed to rotate and translate one of the point clouds so that both of them are under the same coordinate system and the corresponding points are in the same position. The Iterative Closest Points (ICP) algorithm is the most commonly used method for point cloud alignment. It was proposed by Besl [27]. The algorithm is based on the iterative selection of correspondence point relationship sin order to obtain the most suitable transformation parameters. Many studies have proposed improved ICP algorithms for various point alignment tasks. For example, He et al. [28] proposed an improved ICP algorithm based on geometric features, which improves the convergence speed and the interval of convergence without setting a proper initial value. Their algorithm is more suitable for the point clouds of buildings with obvious features. Despite its outstanding advantages, the ICP algorithm is an iterative local minimization method and is sensitive to the initial alignment of the point clouds [29]. Therefore, coarse alignment is usually performed first to obtain a nice initial position, then fine alignment based on the ICP algorithm is performed [28].

Some studies have focused on the alignment of ALS and TLS data. Most studies have implemented the alignment of ALS and TLS point clouds by matching the geometric features of buildings or other human-made objects [30]. Unlike urban areas, which are abundant with distinct geometric features, point cloud alignment in forests has always been a challenge due to the lack of distinct geometric features, and various methods have been developed for point cloud alignment in forest areas [31,32,33].

In this study, the applicability of extracting parameters of individual moso bamboo plants from merged ALS and TLS point cloud data (hereafter referred to as ALS-TLS data) is evaluated. The first objective is to evaluate the quality of the segmentation of individual bamboo plants from the ALS-TLS data and a single data source (ALS or TLS). The second objective is to assess the accuracy of inventory parameters (DBH and height) of individual bamboo plants derived by using the ALS-TLS data and a single data source. To merge the ALS and TLS data, we used a semi-automated point cloud alignment method, i.e., first, a coarse alignment of ALS and TLS data using targets established on the plot corners that can be manually identified from the TLS point cloud and have known coordinates, then a fine alignment using the ICP algorithm. We utilized the point cloud segmentation (PCS) algorithm [18] and the comparative shortest-path (CSP) method [20] to segment individual bamboo plants and extract their heights from ALS, TLS, and ALS-TLS data sets. Then, we used the cylinder fitting method to extract the DBH of individual moso bamboo plants. The accuracies of the parameters of moso bamboo plants retrieved using the three data sets were then examined, using the field survey data as the true values. The rest of this article is structured as follows. Section 2 presents the study area and data acquisition. Section 3 introduces the point cloud data alignment algorithm and the moso bamboo parameter extraction method. Section 4 presents the results. Section 5 and Section 6 present a discussion and conclusions.

## 2. Study Area and Data Acquisition

### 2.1. Study Area

**Figure 1:**
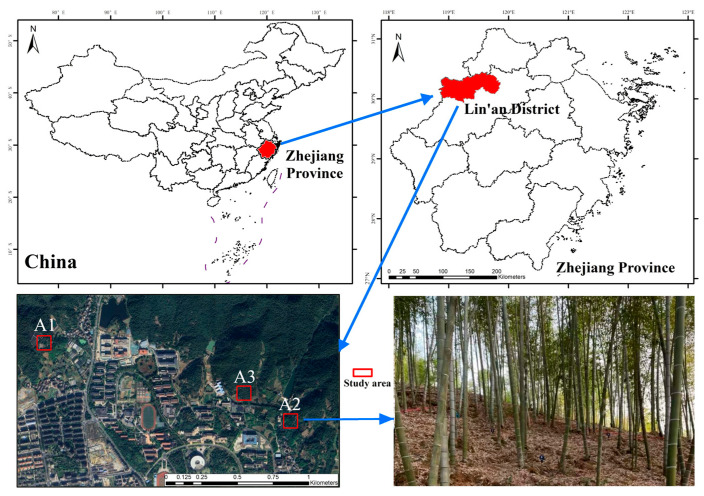
Locations of three moso bamboo sample plots (red rectangles).

The test site discussed in this paper is located in Lin’an District, Hangzhou City, Zhejiang Province, China. It is located at 119°40′ east longitude and 30°15′ north latitude, as shown in Figure 1. The region has a subtropical monsoon climate, with an annual average temperature of 16.4 °C, annual precipitation between about 1500.0 mm and 1628.6 mm, an annual sunshine duration of 1847.3 h, an annual frost-free period of 237 days, and an altitude between 60 m and 120 m. The research object is the commercial moso bamboo forest within Lin’an. The forest mainly contains moso bamboo plants of different ages, from 1 to 4 years. Its site density ranges from about 1500 to 5500 moso bamboo plants per square hectare, and there are few weeds and shrubs in the forest. There are no other tree species in the forest. The test site is hilly terrain, and the soil is slightly acidic red soil.

### 2.2. Data Acquisition

The test site consists of three 10 m × 10 m sample plots (A1, A2, and A3) with standing bamboo densities of 3700 per hm^2^, 4500 per hm^2^, and 5200 per hm^2^, respectively. The bamboo age, DBH, and height were manually surveyed and recorded for each moso bamboo plant. When measuring the height with a Blume-Leiss hypsometer, the highest point of the bamboo crown was observed from a suitable distance, and if there was any obstruction, the other bamboo plants were manually pulled away in order to clearly see the top of the bamboo crown. We set up targets on the four plot corners and obtained the corner coordinates and the position of each bamboo plant by using total stations and real-time kinematics (RTK-GNSS).

**Figure 2:**
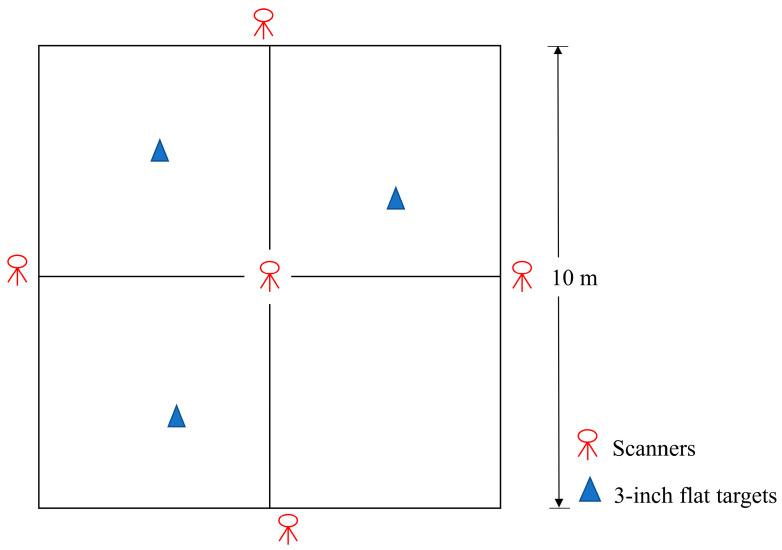
Scanner and target locations in a plot.

The TLS point cloud data were obtained using a Leica Scan Station C05 (Leica Geosystems, Heerbrugg, Switzerland) with a 360° horizontal field of view, a 270° vertical field of view, a scanning rate of 50,000 points per second, and a standard deviation of less than 4 mm within 100 m. The scanner was set up at five scan positions, namely one at the center and four outside the plot. During scanning, we placed three targets evenly in the plot area. The target size was 3 inches. The scanner and target locations are shown in Figure 2.

**Figure 3:**
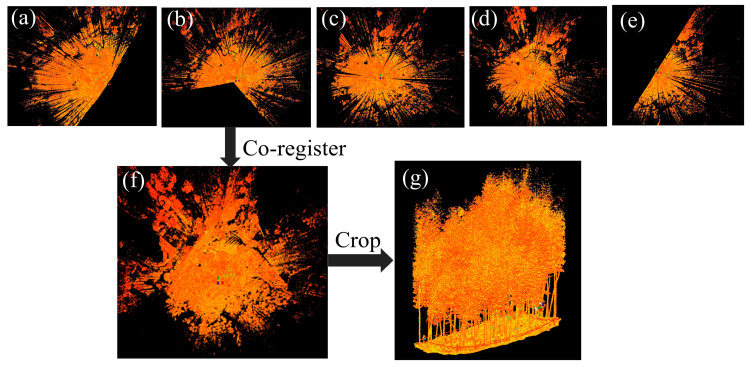
Co-registration and cropping of the TLS data in a sample plot. (**c**) Point cloud captured at the central scan position; (**a**,**b**,**d**,**e**) point clouds captured at the scan positions on the plot edges; (**f**) co-registered point cloud; (**g**) cropped point cloud.

To improve the efficiency of TLS data acquisition and reduce the size of the data, we adopted different scanning modes at different scan positions (see Figure 3). We used a fast scan mode that allowed for the definition of the scan range at the scan positions outside the plot (e.g., Figure 3a) and a panoramic scan mode at the central scan position (e.g., Figure 3c). We preprocessed the TLS data in Leica Cyclone 2024.0.1 software. The co-registration module of Cyclone was used to co-register the raw point cloud data acquired from five scan positions into a point cloud using the three flat targets, and the co-registration error was below 5 mm. The editing module was used to remove noise. For the convenience and efficiency of the subsequent point alignment, the co-registered point cloud data were roughly cropped based on the targets established at the four corners of the plot. The co-registration of the raw point cloud data acquired from five scan positions and the roughly cropped TLS point cloud data are shown in Figure 3.

**Figure 4:**
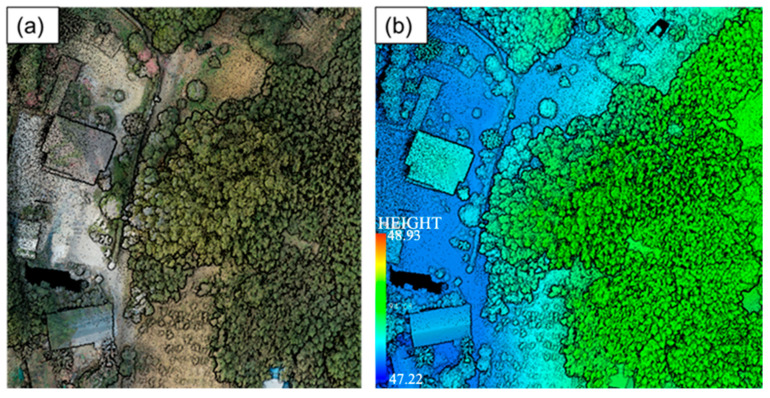
ALS point cloud. (**a**) RGB point cloud; (**b**) elevation-rendering point cloud.

We obtained ALS point cloud data using a DJI Matrice 300 RTK (DJI Innovations, Shenzhen, China) carrying a scanner, the Zenmuse L1 (DJI Innovations, Shenzhen, China). The scanner integrates a Livox LiDAR module (Livox Technology Company Limited, Shenzhen, China), a high-precision IMU, and a camera. The L1 can obtain ground data even in moderate vegetation. The DJI Matrice 300 RTK supports terrain-follow flight. The flying altitude was set to 80 m, the flying speed was 3 m/s, the side overlap was set to 20%, the sampling frequency was 160 kHz, and the average point density was 600 pts/m^2^. The ALS point cloud data acquired over a sample plot are shown in Figure 4. The ALS point cloud data are in the China geodetic coordinate system 2000 (CGCS2000).

## 3. Methodology

### 3.1. Point Cloud Alignment

Since the TLS point cloud data are in a local coordinate system and the ALS data are in the coordinate system of CGCS2000, we needed to convert the TLS point cloud data to the same coordinate system as the ALS point cloud data so that they could be combined into a point cloud. In each plot, we identified the four corner targets from the TLS point cloud data, then performed coordinate transformation using the coordinates of the corner targets measured in the CGCS2000 coordinate system. In dense forests, the RTK usually cannot obtain fixed solutions, and relatively large errors may be contained in the measurements, which affect the coordinate system transformation based on the RTK measurements. In order to further improve the accuracy of point cloud alignment, the ICP algorithm was then used for fine alignment. More details are given as follows.

#### 3.1.1. Coordinate System Transformation

The coordinate system transformation of the TLS point cloud data was performed using the Helmert transformation model, which is frequently used for datum transformation in geodesy. The formula of the model is expressed as follows [34,35]:

(1) $$\left[\begin{array}{c} X\\ Y\\ Z \end{array}\right]=\left[\begin{array}{c} \Delta X\\ \Delta Y\\ \Delta Z \end{array}\right]+\left(1+m\right)\left[\begin{array}{ccc} 1 & \theta_{z} & -\theta_{y}\\ -\theta_{z} & 1 & \theta_{x}\\ \theta_{y} & -\theta_{x} & 1 \end{array}\right]\left[\begin{array}{c} X_{0}\\ Y_{0}\\ Z_{0} \end{array}\right]$$

where Δ*X*, Δ*Y*, and Δ*Z* are translation parameters; *θ_X_*, *θ_Y_*, and *θ_Z_* are rotation parameters; *m* is the scale parameter; [*X*_0_, *Y*_0_, *Z*_0_]′ is the vector of initial coordinates (i.e., coordinates in a local datum); and [*X*, *Y*, *Z*]′ is the vector of transformed coordinates (i.e., CGCS2000 coordinates). The seven transformation parameters were derived using the least squares method based on the two sets of coordinates of the four corner targets in each plot. The established transformation model was then applied to the entire TLS point cloud to obtain the transformed data in the CGCS2000 coordinate system.

#### 3.1.2. Fine Alignment

An efficient matching technique built on the least squares method is the ICP algorithm [27]. Its basic principle is to repeatedly choose corresponding points from the reference point cloud (P1) and the point cloud to be aligned (P2) and calculate the rotation parameter (R) and translation parameter (T) by minimizing the objective function value in Equation (2) as follows:

(2) $$f(R,T)=\frac{1}{n}{\sum_{i=1}^{n}\left\|RP_{i}+T-P_{i}^{\prime }\right\|}^{2}$$

where *R* is the rotation parameter, *T* is the translation parameter; *n* is the number of corresponding point pairs, $P_{i}$ is a point in the airborne point cloud data, and $P_{i}^{\prime }$ is the nearest TLS point corresponding to $P_{i}$.

**Figure 5:**
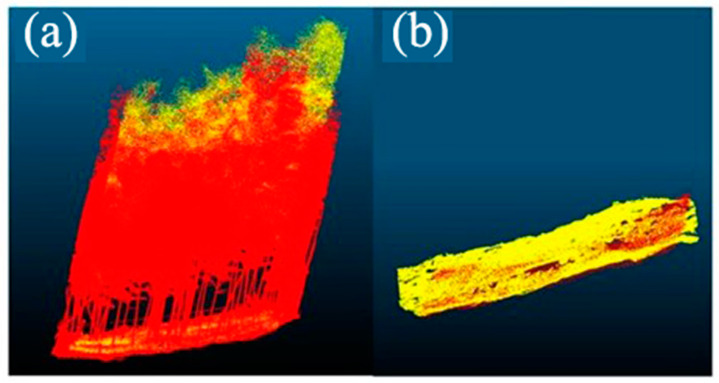
Alignment of TLS point cloud (red) and ALS point cloud (yellow) in a moso bamboo sample plot. (**a**) Aligned point cloud; (**b**) aligned ground point cloud.

The ICP algorithm was implemented in CloudCompare 2.13.1 software. The TLS and ALS data in each moso bamboo sample plot were aligned, and the alignment result, i.e., the ALS-TLS data, is shown in Figure 5. The root mean square error (i.e., root mean square distance between the transformed TLS and ALS point clouds) after fine alignment was 9 mm.

The merged point cloud data should be normalized to eliminate the elevation effects on the following individual bamboo segmentation and parameter extraction. First, we used the Improved Progressive Triangulated Irregular Network Densification (IPTD) [36] algorithm to extract ground points from the ALS-TLS point cloud. Then, a digital elevation model (DEM) with 0.5 m resolution was generated based on the ground points, and the ALS-TLS point cloud was normalized by subtracting the corresponding DEM values from the elevations of the point cloud. Compared with the classical Progressive Triangulated Irregular Network Densification (PTD) algorithm, the IPTD algorithm is able to preserve the terrain features, whereas the PTD algorithm tends to flatten the terrain in areas with steep slopes [37].

### 3.2. Moso Bamboo Parameter Extraction

#### 3.2.1. Single Bamboo Segmentation and Bamboo Height Extraction

The segmentation of individual bamboo plants from point cloud data is a prerequisite for extracting bamboo parameters at the individual bamboo level. Based on the point distribution characteristics of the ALS and TLS data in moso bamboo forests, we used the PCS method [18] to segment individual moso bamboo plants from ALS point cloud data, while the CSP method [20] was used for both TLS and ALS-TLS point cloud data. Both segmentation algorithms were applied to the normalized point clouds.

**Figure 6:**
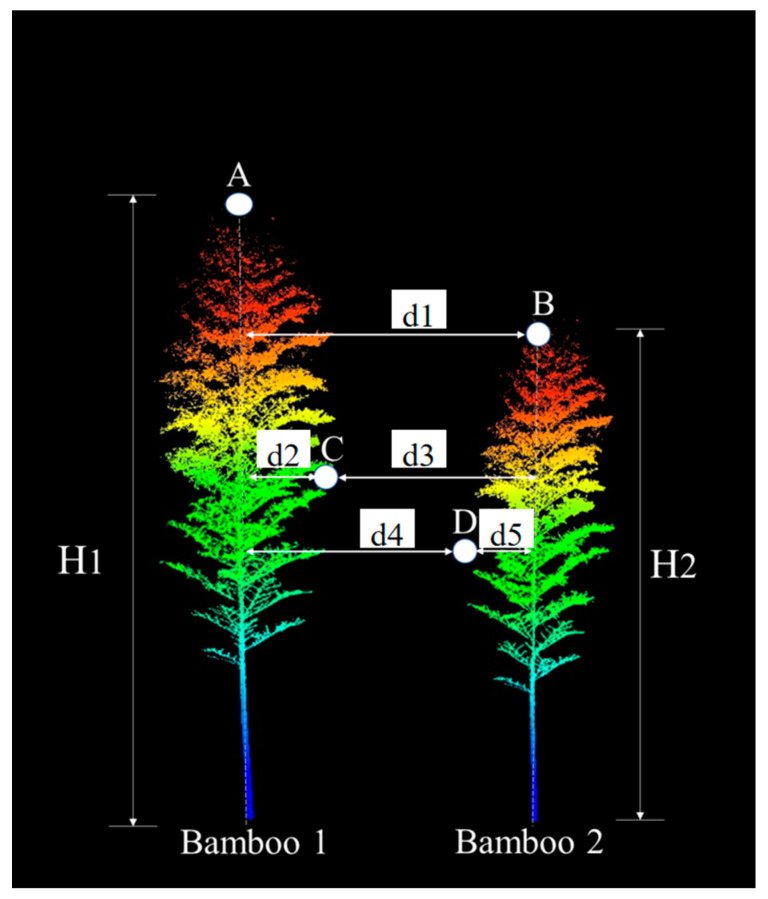
Bamboo segmentation using the PCS algorithm.

The principle of the PCS algorithm is to use the fact that there is a certain distance between bamboo crowns, especially between bamboo tops. The process is to first normalize the point cloud data, then select the highest point as the starting point, consider this point as the vertex of the first bamboo plant, and perform the region-growing algorithm from that point to segment a moso bamboo plant. By iterating such a process, one moso bamboo is segmented each time until all moso bamboos are segmented. The segmentation principle is shown in Figure 6, assuming that Bamboo 1 is the target moso bamboo and the highest point (A) is the vertex of Bamboo 1. Based on the horizontal distances from vertex A, the surrounding points (e.g., points B, C, and D) are assigned to Bamboo 1 or other bamboo plants from top to bottom. If the distance between a point and vertex A is greater than a pre-defined threshold (*d*, defined based on the average spacing of moso bamboo plants in each plot), the point is considered to belong to other bamboo plants. For example, since d1 is greater than the threshold *d*, point B belongs to Bamboo 2, and it is the vertex of Bamboo 2. Point C belongs to Bamboo 1 because d2 is less than d3. Point D belongs to Bamboo 2 because d4 is greater than d5. By repeating these steps, Bamboo 1 is gradually segmented. In the figure, H1 and H2 (the vertical distance between a vertex and the ground) are the heights of the moso bamboo plants.

The CSP method [20,22] is composed of two main steps, i.e., trunk detection and canopy segmentation. First, the density-based spatial clustering of applications with noise (DBSCAN) was used to detect moso bamboo culms. The minimum number of points was set to 500, and the neighborhood radius was set to 0.2 m. The neighborhood radius was defined based on the point density of TLS and ALS-TLS data. Second, the bamboo crown points were assigned to each culm based on the shortest transportation path from each point to the bamboo culms. In order to improve the segmentation accuracy, following the method reported in [22], we applied a second CSP segmentation to the first segmentation result to obtain the final segmentation result. Finally, the culm positions were extracted from the segmentation result, and the vertical distance from the highest point in each bamboo segment to the ground was regarded as the bamboo height.

#### 3.2.2. DBH Extraction

DBH is one of the important tree parameters in forestry. According to the analysis of ALS data, the obscuration of the moso bamboo canopy caused missing data in the bamboo culms, making ALS data not applicable for directly extracting moso bamboo DBH. Therefore, we did not use the ALS data to estimate DBH but only used the TLS and ALS-TLS data. The DBH of moso bamboo plants was extracted using the cylindrical fitting method.

**Figure 7:**
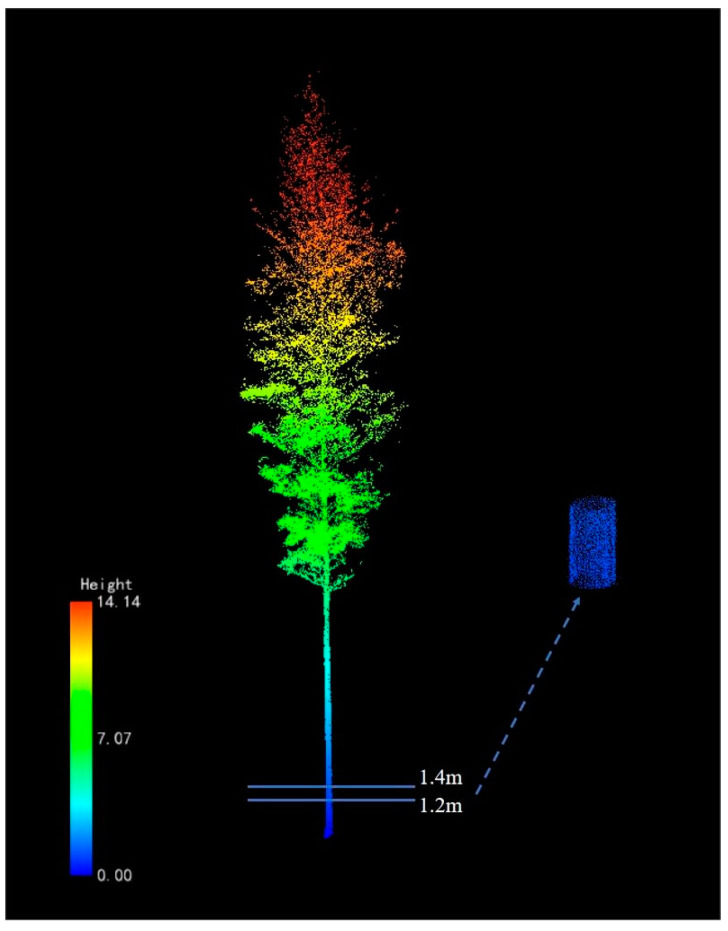
A segment of a bamboo culm point cloud used for cylinder fitting and DBH estimation.

A segment of the bamboo culm point cloud that was extracted between 1.2 and 1.4 m in height (Figure 7) was fitted with a cylinder for each bamboo plant using the least squares method, and the DBH was then calculated [38]. The function of the fitted cylinder is shown as follows:

(3) $$d\left(s,p\right)=\left|(p-(p+\frac{1}{k})\times n)\times a\right|-\frac{1}{k}$$

where ${p=(x,y,z)}^{T}$ is any data point in the three-dimensional point cloud space, *s* is an estimate of the unknown standard deviation of the residuals, *a* is the axial direction of the cylinder, *n* is the normal vector of an orientation of the cylinder, *k* is the reciprocal of the radius of the cylinder, and *d* is the distance between point *p* and the surface of the fitted cylinder.

The nonlinear least squares method was used to minimize the objective function in Equation (4), i.e., the sum of squares of the distances between each point and the cylindrical surface.

(4) $$\min\sum_{i}d^{2}\left(s,p_{i}\right)$$

#### 3.2.3. Accuracy Evaluation

(1) Accuracy evaluation of single bamboo segmentation

The accuracy of individual moso bamboo segmentation is assessed by three parameters, which are recall, precision, and overall accuracy. The recall (*r*) is the detection rate of moso bamboo, (*p*) is the precision of moso bamboo detection, and the *F* score is the overall accuracy [39]. These parameters are calculated using Equations (5) to (7).

(5) $$r=\frac{N_{t}}{N_{t}+N_{o}}$$

(6) $$p=\frac{N_{t}}{N_{t}+N_{c}}$$

(7) $$F=\frac{2\left(r\times p\right)}{r+p}$$

where *N_t_* is the number of correctly segmented bamboo plants, *N_c_* is the number of commission errors, and *N_o_* is the number of omission errors. A segment is considered correct when the detected bamboo location (i.e., the highest point within the segment) is located within a distance from a reference measured in the field. The distance threshold was defined according to the average spacing of bamboo in each plot. A bamboo plant that actually exists but is not segmented by the algorithm is regarded as an omission error. A segment that does not correspond to any reference is regarded as a commission error [40].

(2) Accuracy evaluation of single bamboo parameter extraction

The coefficient of determination (R^2^) and root mean square error (RMSE) were computed to evaluate the accuracy of the extracted DBH and the heights of the correctly segmented bamboo plants. We derived R^2^ through a linear regression analysis of the relationship between the extracted parameter values (i.e., DBH and bamboo height) and the measurements obtained in the field. The RMSE values were calculated using the following equation:

(8) $$\mathrm{RMSE}=\sqrt{\frac{\sum_{i=1}^{n}{\left(y_{i}-y_{i}^{\prime }\right)}^{2}}{n}}$$

where *n* is the number of moso bamboo plants, $y_{i}^{\prime }$ is the DBH or bamboo height measured in the field, and *y_i_* is the extracted DBH or bamboo height for a segmented moso bamboo plant. The accuracies were statistically analyzed in Excel and Origin 2023 software.

## 4. Results

### 4.1. Single Bamboo Detection

**Figure 8:**
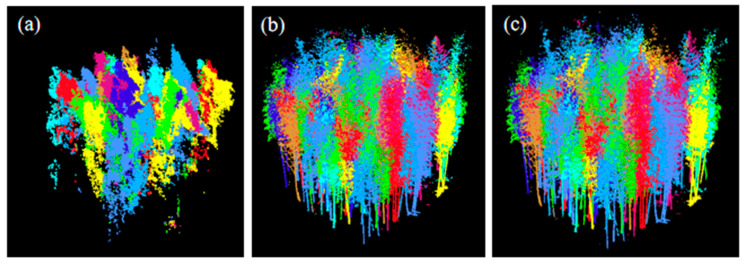
Moso bamboo segmentation from (**a**) ALS point cloud, (**b**) TLS point cloud, and (**c**) ALS-TLS point cloud. Different colors distinguish the different moso bamboo plants.

The bamboo segmentation results obtained by applying the PCS and CSP algorithms in sample plot A2 using different data sets are shown in Figure 8. As shown in the figure, the ALS point cloud data have an obvious lack of laser points in the bamboo culm layer due to obstruction by the dense canopy.

**Figure 9:**
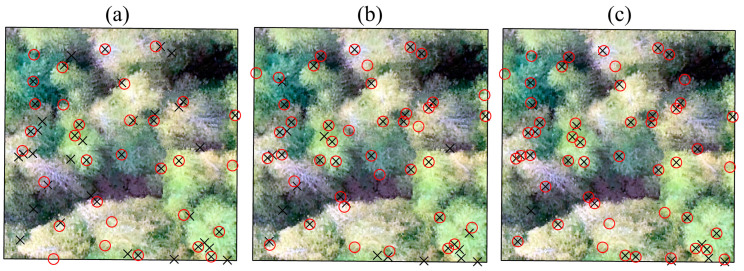
Moso bamboo detection results derived using (**a**) ALS data, (**b**) TLS data, and (**c**) ALS-TLS point cloud.

Figure 9 shows the detected bamboo plant locations and the reference locations collected in the field. As shown in Figure 9, when applying the PCS algorithm to the ALS data, the bamboo plants that were close to each other could not be correctly segmented, and under-segmentation errors tended to occur.

Table 1 shows the accuracy of moso bamboo segmentation using TLS, ALS, and ALS-TLS data in three sample plots. The value of *r* varies from 55.77% to 97.30%, the value of *p* varies from 76.32% to 92.59%, and the value of *F* varies from 0.64 to 0.94. In dense plots, bamboo plants tend to be under-segmented, and the value of r is relatively low. For example, the values of *r* obtained in sample plot A3 based on all three data sources are smaller than those in sample plots A1 and A2, and more bamboo plants were undetected in plot A3, especially when using the ALS data. In contrast, higher values of *r* and *P* were derived in sample plot A1 than in the other two plots. Moreover, the value of *r* in sample plot A1 based on ALS-TLS data is 97.30%, indicating fewer omission errors. In brief, using the ALS-TLS data derived higher recall, precision, and *F*-score values than using a single data source (TLS or ALS). The recall values derived by using ALS data are much lower than those derived by using TLS data, indicating the difficulty in segmenting the interlacing bamboo crowns. In contrast, the difference in precision values among the three data sets is small in comparison with the difference in recall values. Over-segmentation errors tended to occur when using both segmentation algorithms.

### 4.2. Accuracy of Single Bamboo Parameter Extraction

#### 4.2.1. Accuracy of DBH Estimation

**Figure 10:**
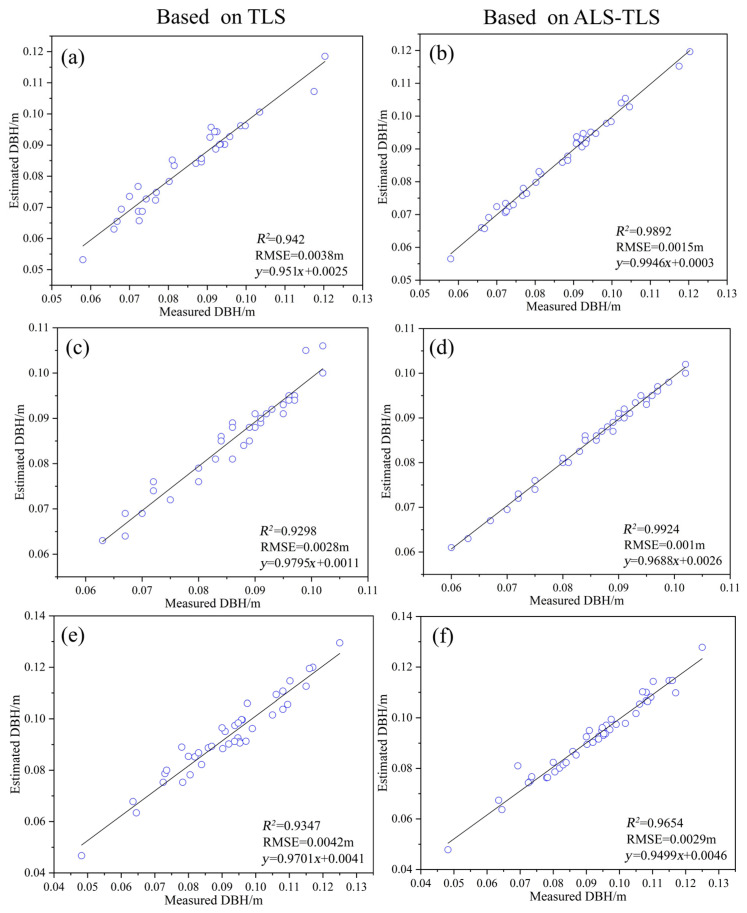
DBH estimation by cylindrical fitting based on different data sources. (**a**,**b**) Plot A1; (**c**,**d**) plot A2; (**e**,**f**) plot A3.

Due to the relatively sparse bamboo culm points of ALS data, only the TLS and ALS-TLS data were compared for DBH estimation. By fitting a linear regression model between the estimated DBH and the DBH measured in the field, the accuracy of the estimated DBH was examined. The accuracy of DBH estimation using cylindrical fitting with TLS and ALS-TLS data in sample plots A1, A2, and A3 is shown in Figure 10. In all three sample plots, the accuracies of DBH estimation based on ALS-TLS data are higher than those derived based on TLS data. The R^2^ of DBH estimation based on TLS data ranges from 0.93 to 0.94, and the RMSE ranges from 0.003 m to 0.004 m. The R^2^ of DBH estimation based on ALS-TLS data ranges from 0.97 to 0.99, and the RMSE ranges from 0.001 m to 0.003 m. The results show that although the accuracy of DBH estimation based on TLS data is high, points are missing on the bamboo culms due occlusion by branches and leaves, which affects the estimation of DBH. Using the ALS-TLS data, we can obtain more complete point cloud data on the bamboo culms and, hence, derive a more accurate DBH estimation.

#### 4.2.2. Accuracy of Bamboo Height Extraction

**Figure 11:**
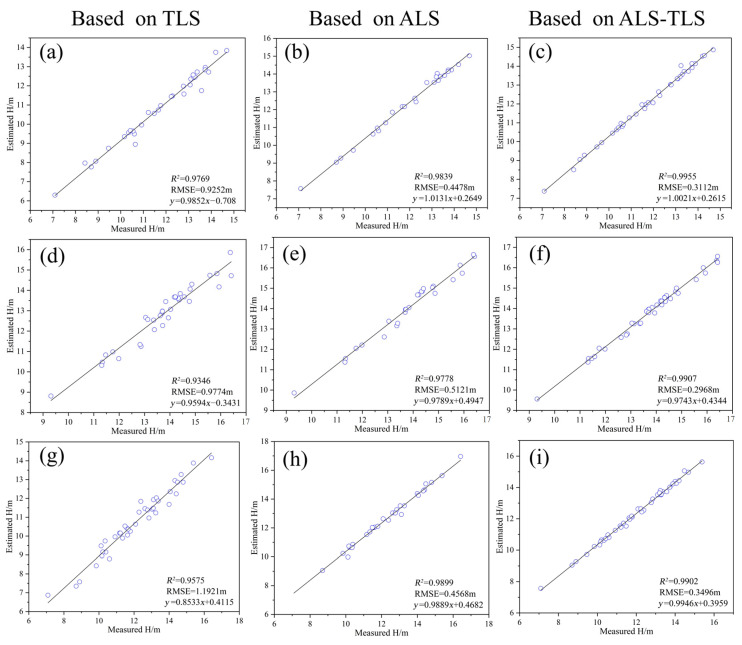
Bamboo height extraction based on different data sources. (**a**–**c**) Plot A1; (**d**–**f**) plot A2; (**g**–**i**) plot A3.

In Figure 11, the bamboo heights extracted based on TLS, ALS, and ALS-TLS data are compared against the measured bamboo heights in sample plots A1, A2, and A3, respectively. It can be observed that the bamboo heights extracted based on ALS-TLS data have the highest accuracies, while the accuracies derived using TLS data are the lowest. The R^2^ of bamboo height extracted based on TLS and ALS data in the three sample plots ranges from 0.92 to 0.96 and 0.94 to 0.98, and the RMSE ranges from 0.98 m to 1.14 m and 0.63 m to 0.80 m, respectively. The R^2^ of bamboo height extracted based on ALS-TLS data is higher than 0.96, and the RMSE ranges from 0.35 m to 0.48 m. The much lower accuracies derived using TLS data are due to the bottom-up scanning mode of TLS. The upper part of the bamboo canopy only has sparse or even no points, leading to poor accuracy of bamboo height estimation. Moreover, due to the overlapping moso bamboo crowns and the bottom-up scanning mode of TLS, it is difficult to find the actual bamboo tops, and the points of a neighboring bamboo plant may be mistakenly regarded as the top of the target bamboo plant, resulting in an overestimation or underestimation of bamboo height. The ALS-TLS data integrate the ALS and TLS data; thus, the moso bamboo canopy point cloud is more complete, which is beneficial for improving the accuracy of bamboo height extraction.

## 5. Discussion

### 5.1. Point Cloud Alignment

The alignment of the TLS and ALS point cloud data is an important step for subsequent individual bamboo segmentation and parameter extraction. Much attention should be paid to this step to eliminate the discrepancy between the transformed TLS and the ALS point cloud data. The ICP algorithm has been commonly used for the alignment of TLS and ALS point clouds in recent studies [33]. It can effectively reduce the influence of a small amount of noise on the alignment and has certain advantages in terms of speed and accuracy. However, the requirement of the ICP algorithm for the initial position of the corresponding points is high. Moreover, unlike in urban areas, it is difficult to find distinct features for point cloud alignment in forest areas. To solve this problem, we first performed a coordinate transformation on the TLS point cloud data based on the measured coordinates of plot corner targets so that the two point clouds were in a better initial position, then applied the ICP algorithm to refine the point cloud alignment result. Actually, if the measurements of the plot corner targets are accurate enough, the TLS and ALS point clouds can be directly combined to obtain a merged point cloud. However, in dense forests, the GPS signal is usually weak, leading to poor coordinate accuracy, which affects the accuracy of point cloud alignment. Therefore, we applied the ICP algorithm following the coordinate transformation of the TLS point cloud data. In a further study, we will evaluate the applicability of such a strategy to various forest types.

### 5.2. Parameter Extraction

Moso bamboo is a monopodial and clumping bamboo species that has true underground stems (rhizomes), whereas the stems on the ground grow in a scattered manner. During growth, moso bamboo plants may be close to each other, causing overlapping crowns. Due to the top–bottom scanning mode, the point density of ALS data decreases from the upper to the lower part of the canopy layer. Using such data, under-segmentation and over-segmentation tend to occur. In contrast, the TLS technique scans from the ground, and the bamboo culms have dense laser points. Due to the lack of understory vegetation, it is much easier to detect bamboo culms than to segment individual crowns. However, there may be fallen moso bamboo plants in the forest, which cause different degrees of occlusion and influence the detection of moso bamboo plants. In this article, we used the PCS and CSP algorithms to extract individual moso bamboo plants and bamboo heights. The bamboo plants that were close to each other could not be correctly segmented from the ALS point cloud data using the PCS algorithm, and much lower detection rates were derived. To eliminate the influence of the segmentation errors, we only analyzed the accuracy of the extracted parameter values of the correctly segmented bamboo plants.

The detection rate of moso bamboo based on ALS-TLS data is higher than the detection rate obtained by Huang et al. [41], who detected moso bamboo based on TLS data. Our results also show that the accuracy of the extracted bamboo height based on ALS-TLS data is higher than the accuracy derived by using a single data source. The reason is that the combination of TLS and ALS data can generate a more complete point cloud of the bamboo forest. The result is consistent with the findings of Tian et al. [42] with respect to height extraction in medium- and high-density planted conifer forests using a combination of TLS and unmanned aerial vehicle (UAV) image-based point cloud data.

In this article, we used the cylindrical fitting method for DBH estimation. Compared with the two-dimensional curve fitting method, the DBH accuracy obtained by the cylinder fitting method is higher. This is because the cylindrical fitting method directly models the points on the bamboo culm surface. Therefore, the inaccuracy caused by point cloud alignment is reduced, and more bamboo culm features are retained. By applying this method, high DBH estimation accuracies were derived for both TLS and ALS-TLS data. Due to the more complete point cloud of the bamboo culms, higher DBH estimation accuracies were derived by using the ALS-TLS data. The segmentation results of the fused point cloud in Table 1 show improvements in both recall and precision, indicating that point cloud fusion enhances the accuracy and precision of bamboo segmentation. This improvement consequently affects the accuracy of subsequent parameter extraction.

### 5.3. Limitations

Since the main objective of this study is not to develop a bamboo segmentation or parameter extraction method, we used commonly used methods to segment individual bamboo plants and extract their parameters. Deep learning has been applied to individual tree segmentation and individual tree parameter estimation [43,44]. Due to the small number of sample plots in this study, the sample size is insufficient to meet the large training data requirements for deep learning. In further research, other methods, including deep learning methods, can be used for individual bamboo plant segmentation and parameter extraction to improve accuracy.

It should be noted that acquiring both TLS and ALS data will cost more time and money, and the acquisition of TLS data is usually limited to a few sample plots. However, accurate inventory parameters of moso bamboo acquired by using both data sources in sample plots can be used to estimate the volume and biomass in the plots and can be further used for the construction of a biomass estimation model in the larger area covered by the ALS data or other remote sensing data [5,6,7].

## 6. Conclusions

In this article, focusing on a moso bamboo forest, an approach for aligning the ALS and TLS data and estimating individual bamboo parameters based on the merged point cloud is proposed. When aligning TLS and ALS data, the TLS point cloud was first transformed to the same coordinate system as the ALS point cloud using the Helmert transformation model. Then, the ICP algorithm was applied to perform the fine alignment of the TLS and ALS point clouds. A millimeter-level accuracy of alignment was achieved.

The accuracy of moso bamboo detection derived based on ALS-TLS data (*F* score between 0.82 and 0.94) was higher than that based on TLS (*F* score between 0.77 and 0.88) or ALS data (*F* score between 0.64 and 0.78). The error of the bamboo height estimated based on TLS data was the largest, with an RMSE as high as 1.14 m. The bamboo height extracted from ALS-TLS data was closer to the measured value, with an RMSE of less than 0.48 m. The RMSE values of the moso bamboo DBH estimated by using the cylindrical fitting method based on TLS and ALS-TLS data were in the ranges of 0.003~0.004 m and 0.001~0.003 m, respectively. The results show that although high accuracies were derived for both data sets, the DBH estimation accuracies derived based on the ALS-TLS data were higher due to the more complete point cloud of bamboo culms, indicating the advantage of the merged point cloud.

In conclusion, the moso bamboo parameters extracted based on ALS-TLS data are closer to the measured values than those extracted based on single data sources, indicating the advantage of the merged point cloud and the applicability of the proposed approach for point cloud alignment and parameter extraction. Although acquiring both TLS and ALS data increases time requirements and cost, the accurately estimated inventory parameters of moso bamboo in sample plots can be used to construct a biomass estimation model in a larger area covered by ALS data or other remote sensing data.

## Figures and Tables

**Table 1:** Detection of moso bamboo in the sample plots (*n*: actual number of bamboo plants; *N_t_*: the number of correctly segmented bamboo plants; *N_c_*: the number of commission errors; *N_o_*: the number of omission errors; *r*: the detection rate of bamboo plants; *p*: the precision of bamboo detection; *F*: *F* score).

Plot ID	*n*	Data Sources	Number of Segmented Bamboo Plants	*N_t_*	*N_c_*	*N_o_*	*r*	*p*	*F*
A1	37	ALS	27	25	2	12	67.57%	92.59%	0.78
		TLS	36	32	4	5	86.49%	88.89%	0.88
		ALS-TLS	40	36	4	1	97.30%	90.00%	0.94
A2	45	ALS	32	26	6	19	57.78%	81.25%	0.68
		TLS	42	35	7	10	77.78%	83.33%	0.80
		ALS-TLS	50	42	8	3	93.33%	84.00%	0.88
A3	52	ALS	38	29	9	23	55.77%	76.32%	0.64
		TLS	52	40	12	12	76.92%	76.92%	0.77
		ALS-TLS	55	44	11	8	84.62%	80.00%	0.82

## Data Availability

The data presented in this study are available upon request from the corresponding author.
